# CAMIL: channel attention-based multiple instance learning for whole slide image classification

**DOI:** 10.1093/bioinformatics/btaf024

**Published:** 2025-01-16

**Authors:** Jinyang Mao, Junlin Xu, Xianfang Tang, Yongjin Liu, Heaven Zhao, Geng Tian, Jialiang Yang

**Affiliations:** School of Mathematics and Statistics, Fuzhou University, Fuzhou 350108, China; School of Computer Science and Technology, Wuhan University of Science and Technology, Hubei 430065, China; School of Computer Science and Artificial Intelligence, Wuhan Textile University, Wuhan 430200, China; School of Mathematics and Statistics, Fuzhou University, Fuzhou 350108, China; Geneis Beijing Co., Ltd, Beijing 100102, China; Geneis Beijing Co., Ltd, Beijing 100102, China; Geneis Beijing Co., Ltd, Beijing 100102, China

## Abstract

**Motivation:**

The classification task based on whole-slide images (WSIs) is a classic problem in computational pathology. Multiple instance learning (MIL) provides a robust framework for analyzing whole slide images with slide-level labels at gigapixel resolution. However, existing MIL models typically focus on modeling the relationships between instances while neglecting the variability across the channel dimensions of instances, which prevents the model from fully capturing critical information in the channel dimension.

**Results:**

To address this issue, we propose a plug-and-play module called Multi-scale Channel Attention Block (MCAB), which models the interdependencies between channels by leveraging local features with different receptive fields. By alternately stacking four layers of Transformer and MCAB, we designed a channel attention-based MIL model (CAMIL) capable of simultaneously modeling both inter-instance relationships and intra-channel dependencies. To verify the performance of the proposed CAMIL in classification tasks, several comprehensive experiments were conducted across three datasets: Camelyon16, TCGA-NSCLC, and TCGA-RCC. Empirical results demonstrate that, whether the feature extractor is pretrained on natural images or on WSIs, our CAMIL surpasses current state-of-the-art MIL models across multiple evaluation metrics.

**Availability and implementation:**

All implementation code is available at https://github.com/maojy0914/CAMIL.

## 1 Introduction

Histopathological examination is widely regarded as the gold standard for cancer diagnosis and prognosis in clinical practice (Lu *et al.* 2021a). Historically, pathologists relied on microscopic examination of stained tissue sections to identify tumorous regions. However, with the advent of high-resolution scanning technologies, traditional glass slides have been progressively supplanted by whole-slide images (WSIs) (Cai *et al.* 2024). The advancement in computational capabilities and the rapid emergence of artificial intelligence algorithms have presented new opportunities for analyzing WSIs. Currently, computer vision algorithms have been successfully applied in various cancer clinical research, including tumor identification (He *et al.* 2012, Chen and Srinivas 2016, Yao *et al.* 2022), mutation gene prediction (Coudray *et al.* 2018, Huang *et al.* 2022), and cellular segmentation (Graham *et al.* 2019, Greenwald *et al.* 2022). Unlike conventional natural images, WSIs are characterized by their immense size, typically comprising billions of pixels (e.g. 40 000 × 40 000 pixels) (Srinidhi *et al.* 2021). This distinctive characteristic presents two significant challenges for WSI-based analysis. Firstly, the vast scale of WSIs renders fine-grained annotations (such as pixel-level or patch-level) nearly impossible, as WSI annotation requires specialized expertise and is both time-consuming and labor-intensive, and so slide-level annotations are generally the only available labeling information (Qu *et al.* 2022). Secondly, feeding such large-scale WSIs directly into deep learning models presents a significant challenge to GPUs memory capacity (Fang *et al.* 2024).

Multiple instance learning (MIL), as a form of weakly supervised learning (Wang *et al.* 2020, Dov *et al.* 2021), has been widely adopted in WSI analysis due to its distinctive characteristic of not requiring instance-level labels. Although MIL effectively addresses the lack of fine-grained labels in WSIs, the end-to-end training of MIL models remains challenging due to the extremely high computational cost (Tang *et al.* 2024). Consequently, current mainstream deep learning approaches based on MIL adopt a two-stage methodology: (i) Feature extraction: pre-trained CNN or Transformer models are utilized as encoders to extract feature vectors from image patches. (ii) Feature aggregation: instance-level feature aggregator is designed to perform feature aggregation operations on the feature vectors within each bag, resulting in bag-level feature vectors for subsequent downstream prediction tasks. Given that the aggregation operation compresses the information from billions of pixels in WSIs into a single global representation, the design of a robust feature aggregator is crucial for model performance. Attention-based paradigms (Ilse *et al.* 2018, Lu *et al.* 2021b, Shao *et al.* 2021) have been widely adopted for feature aggregation, aiming to train models to assign an attention score to each instance. Despite the impressive achievements of attention-based methods in computational pathology, almost all existing work focuses solely on spatial dimension attention weight allocation (where all values across the channel dimensions within an instance are treated as equally important), neglecting the variability across channel dimensions. Channel attention mechanisms have been demonstrated to enhance models' comprehension of contextual information in natural images and have achieved notable success in various domains, including object detection (Woo *et al.* 2018, Li *et al.* 2021b, Qin *et al.* 2021), classification tasks (Karthik *et al.* 2022, Li *et al.* 2022), and image segmentation (Mou *et al.* 2019, Huang *et al.* 2023, Fu *et al.* 2024). However, these methods have been rarely utilized in pathological image classification.

To address the challenges mentioned above, we introduce CAMIL, a novel model that integrates channel attention mechanisms with transformers for WSI classification. Inspired by TransMIL (Shao *et al.* 2021), we have designed an innovative positional encoding module called the Multi-scale Channel Attention Block (MCAB). This module not only encodes positional information but also captures channel information crucial for downstream tasks. Our approach involves feeding extracted feature vectors into stacked Transformers and the MCAB. The Transformer models inter-instance relationships, while the MCAB explicitly models inter-channel dependencies. Through this design, our CAMIL can simultaneously model both spatial and channel dimensions, enabling it to learn richer information from WSIs. The main contributions of this paper are as follows:

1. We propose a novel multi-scale channel attention mechanism that models inter-channel dependencies using local features with varying receptive fields. Through a series of ablation studies, we provide comprehensive evidence for the performance improvements afforded by the MCAB from multiple perspectives.
2. Channel attention mechanisms are introduced to WSI classification tasks, demonstrating that the integration of channel attention with transformers enhances model performance. To the best of our knowledge, this is the first work to introduce channel attention into WSI classification.
3. We validate the superiority of CAMIL over existing state-of-the-art models through comparative experiments on three datasets. Our work provides new insights into the application of MIL models in the field of pathological image analysis.

## 2 Related work

### 2.1 Multiple instance learning in WSI classification

MIL is a weakly supervised learning method aimed at learning a mapping from a bag of instances to a bag-level label (Maron and Lozano-Pérez 1997). Unlike standard supervised learning frameworks, in MIL, each WSI is viewed as a labeled bag containing a large sequence of unlabeled patches (instances) of the same length. Ilse *et al.* (2018) proposed the attention-based multiple instance learning (ABMIL) model, which uses an attention mechanism to perform weighted averaging of instances within a bag by training an importance parameter for each instance. Lu *et al.* (2021b) proposed the clustering-constrained attention multiple instance model (CLAM), which incorporates instance-level clustering constraints into the attention mechanism. This model not only identifies important regions through attention but also clusters these regions to further refine the feature space, thereby enhancing classification accuracy. Li *et al.* (2021a) proposed an aggregator with masked nonlocal operation, capable of modeling inter-instance relationships using trainable distance measurements. As WSI classification essentially involves identifying the most salient patches relevant to the bag-level label, and the number of bags is significantly smaller than the number of constituent instances, Zhang *et al.* (2022) addressed this imbalance by introducing pseudo-bags to artificially increase the number of bags and reduce model prediction difficulty. Javed *et al.* (2022) presented a simple formulation of MIL that enhances model interpretability and demonstrated that most models could achieve additivity through a simple functional modification. Transformer architecture (Vaswani *et al.* 2017), renowned for their powerful long-range contextual association capabilities, have revolutionized the field of computer vision, including pathological image analysis. For example, Shao *et al.* (2021) observed that previous multi-instance learning research was based on the independent and identically distributed assumption, which they deemed unreasonable. Consequently, they proposed TransMIL, which incorporates the Transformer architecture into multiple-instance learning, modeling inter-instance relationships within the same bag through self-attention. In addition to TransMIL, there are some other Transformer-based approaches (Chen *et al.* 2022, Wang *et al.* 2022, Wagner *et al.* 2023) that have been applied to digital pathology.

### 2.2 Channel attention in deep learning

The attention mechanisms are widely used in natural language processing and computer vision, which play a crucial role in deep learning. The core of attention mechanisms involves learning weight distributions from relevant feature maps, applying these learned weights to the original feature maps, and then performing weighted summation. Unlike traditional attention, channel attention aims to explicitly model interdependencies between channels. Specifically, it learns the importance of each channel and enhances useful features while suppressing less significant ones for the current task. While this inevitably increases the parameter count, the performance improvements generally justify the additional computational cost.

Hu *et al.* (2018) first proposed channel attention with their squeeze-and-excitation network (SE-Net). The SE-Net uses a squeeze-and-excitation block to model inter-channel dependencies, using global average pooling (GAP) to obtain a compressed feature with a global receptive field, followed by an MLP to calculate weights for each channel. Woo *et al.* (2018) introduced the convolutional block attention module (CBAM), which enhances the feature representation capability of CNN through attention mechanisms applied to both channel and spatial dimensions. CBAM's strategy of sequentially applying channel and spatial attention enables networks to capture informative channels while focusing on important regions. Wang *et al.* (2020) argued that capturing relationships between all channels is inefficient and unnecessary. They proposed ECA-Net, replacing the MLP in the SE Block's excitation step with a 1D convolution, significantly reducing parameters while achieving appropriate cross-channel interaction. Qin *et al.* (2021) contended that GAP cannot captures rich input pattern information. Approaching from a frequency domain perspective, they demonstrated that GAP is equivalent to the lowest frequency component of the discrete cosine transform (DCT) and proposed FcaNet with multi-spectral channel attention.

Despite the widespread application of channel attention mechanisms in medical imaging (Mou *et al.* 2019, Ni *et al.* 2020, Zhu *et al.* 2021, Pan *et al.* 2022, Chi *et al.* 2023, Huang *et al.* 2023, Fu *et al.* 2024), they are rarely used in WSIs analysis. A possible reason is the variable number of patches in each WSI, which precludes the use of CNN or pooling operations to obtain channel masks.

## 3 Materials and methods

### 3.1 Dataset description

**Figure 1.**
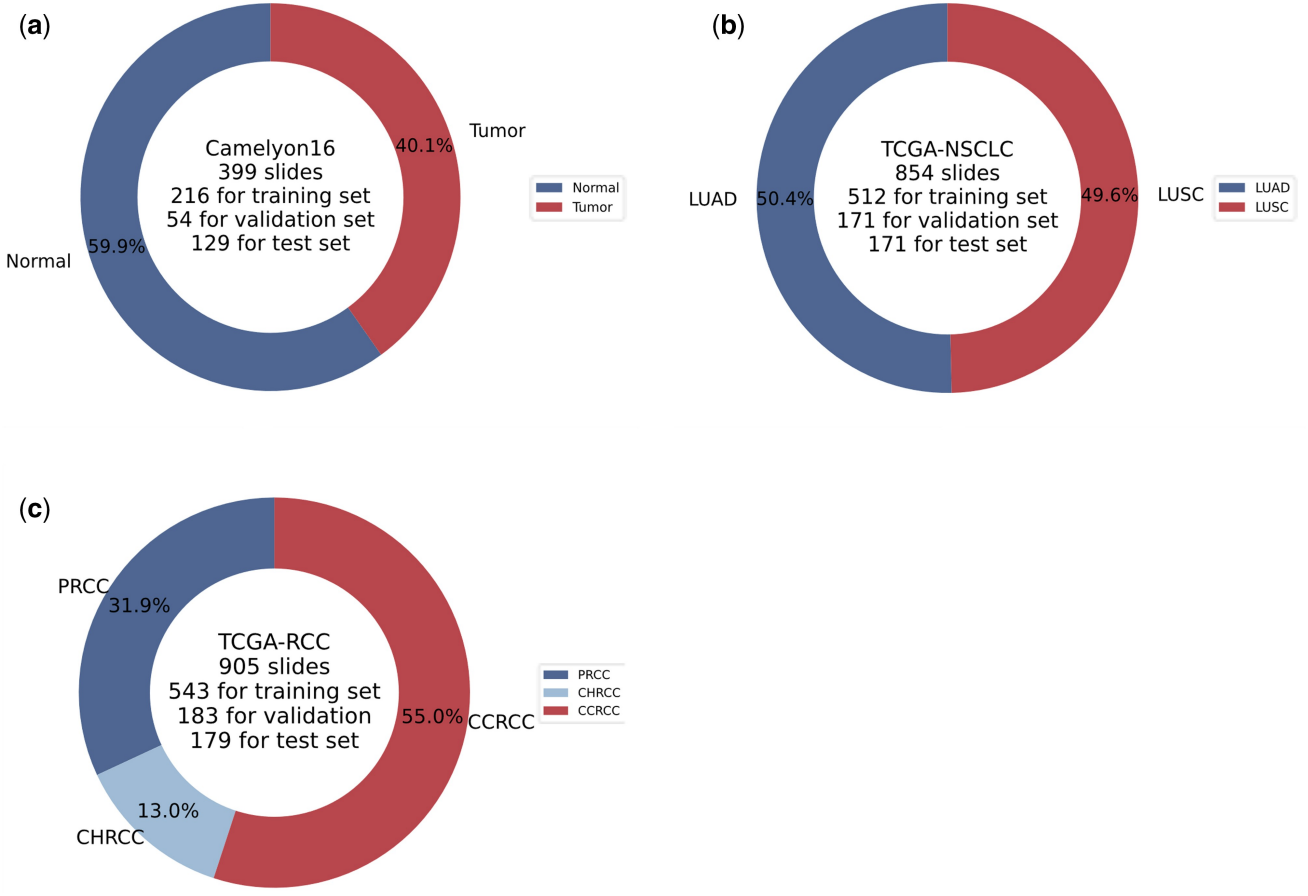
Data distribution of (a) Camelyon16, (b) TCGA-NSCLC, and (c) TCGA-RCC datasets.

Two public datasets were used to validate CAMIL's effectiveness: Camelyon16 (Ehteshami Bejnordi *et al.* 2017) and The Cancer Genome Atlas (TCGA). Cameyon16 is a dataset for a breast cancer recurrence detection challenge. In the official split, it includes a training set of 270 WSIs and a test set of 129 WSIs. To ensure the reliability of the experimental results, we mix these slides and re-split them for further experimentation. As shown in Fig. 1a, the classification ratio is about 6:4, which is close to balance. After preprocessing, we extract a total of 3.6 million nonoverlapping patches, each sized 256 × 256 pixels at 20× magnification. On average, each WSI contains 9067 patches.

The Cancer Genome Atlas (TCGA) is a comprehensive public cancer dataset encompassing over 30 cancer types from multiple institutions. To verify the performance of the model in binary and multi-classification tasks, we utilize datasets from two cancer types: Non-Small Cell Lung Cancer (NSCLC) and Renal Cell Carcinoma (RCC). The TCGA-NSCLC dataset involves two subtypes: Lung Squamous Cell Carcinoma (LUSC) and Lung Adenocarcinoma (LUAD). Figure 1b shows that the distribution of WSIs for the two subtypes of NSCLC is balanced, with a total of 854 WSIs, including 430 LUAD WSIs and 424 LUSC WSIs. After preprocessing, we extracted approximately 11 million nonoverlapping patches at 20× magnification, with each WSI containing an average of 12 860 patches. The TCGA-RCC dataset primarily comprises three subtypes: Papillary Renal Cell Carcinoma (PRCC), Chromophobe Renal Cell Carcinoma (CHRCC), and Clear Cell Renal Cell Carcinoma (CCRCC). Figure 1c shows the distribution of the three RCC subtypes, with a total of 905 WSIs. CCRCC accounts for more than half, with 498 slides, while PRCC and CHRCC have 289 and 118 slides, respectively. After preprocessing, we extracted approximately 13 million nonoverlapping patches at 20× magnification, resulting in an average of 14 771 patches per WSI.

For both Cameyon16 and TCGA datasets, we used a 5-fold cross-validation strategy for data partitioning. From each fold’s training set, we further allocated a validation set to supervise the training process, resulting in a 3:1:1 ratio for training, validation, and test sets, respectively. To prevent information leakage, we ensured that slides from the same patient did not appear simultaneously in both training sets and test sets.

### 3.2 Formulation of multiple instance learning

In the framework of MIL, a bag is composed of a series of instances. In the binary classification scenario, a bag is considered negative when all instances within it are negative, while the presence of at least one positive instance renders the bag positive. In other words, the objective of MIL is to identify whether at least one positive instance exists within a bag. Ilse *et al.* (2018) provided the following definition for bag-level labels:

(1) Yi=0, iff ∑yik=0,yik∈0,11, otherwise

However, only bag-level labels are available, while instance-level labels remain unknown. We can predict the bag label using the following formula:

(2) Y^=h(g(f(xi0),f(xi1),…,f(xik))

where f(·) is an instance-level feature extractor that maps instances to an embedding space. g(·) is an aggregation operation that consolidates instance-level embeddings into a bag-level embedding. h(·) is a classifier that predicts the label for the entire bag based on the specific task.

### 3.3 Framework overview of CAMIL

**Figure 2.**
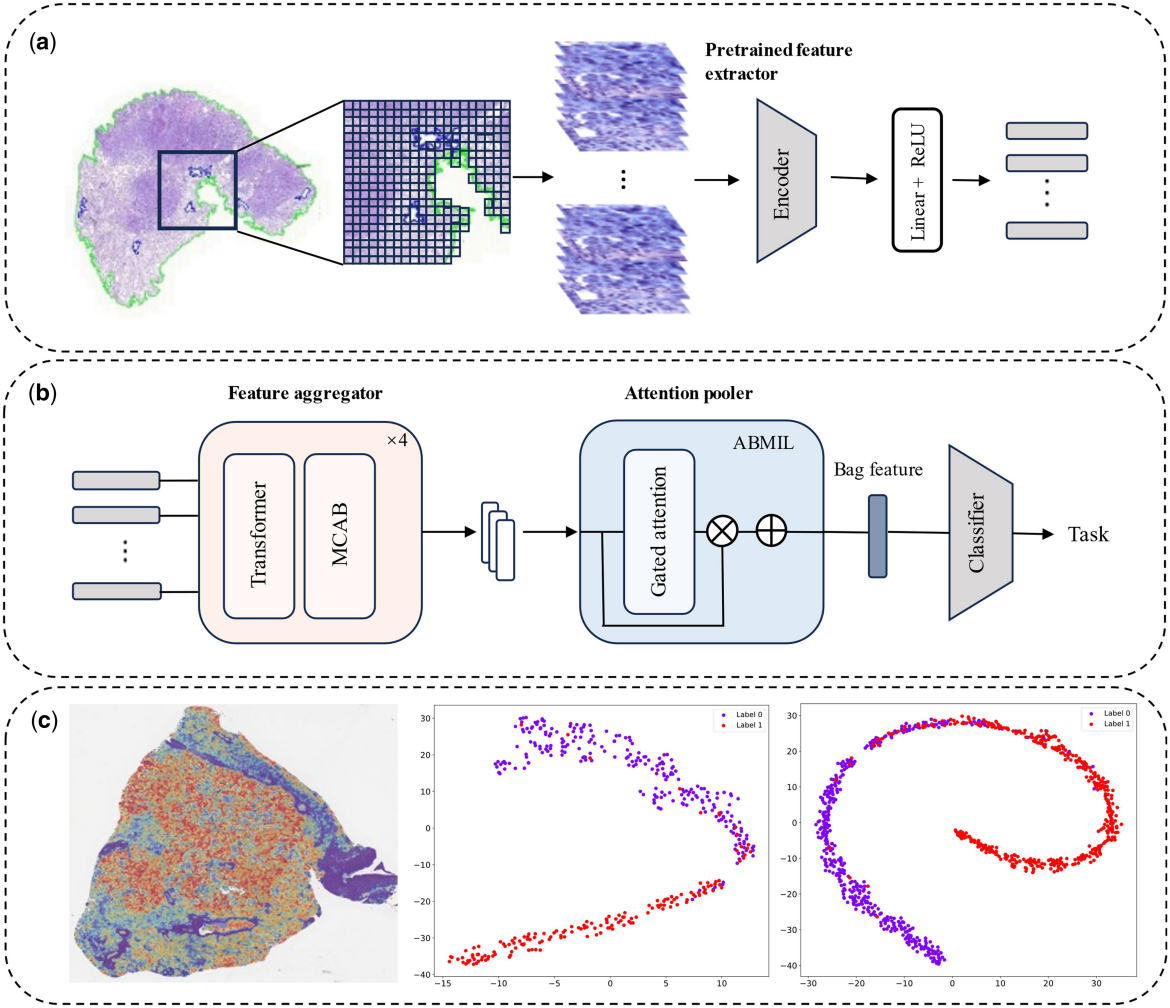
The whole workflow of our CAMIL. (a) The data preprocessing pipeline. The WSI undergoes tissue segmentation to remove background regions. Then segmented image is divided into nonoverlapping patches of 256 × 256 pixels. A pre-trained extractor is used to extract features from these patches and a fully connected layer with ReLU activation is used to reduce the dimensionality of the extracted features. (b) Model architecture. The extracted features are fed into a stack of Transformer modules and the MCAB. (c) Model evaluation. Various visualization tools are used to demonstrate the model’s performance.

As illustrated in Fig. 2, CAMIL consists of three main components: instance-level feature extraction, bag-level feature generation, and classification. Initially, WSIs are segmented into nonoverlapping patches of 256 × 256 pixels. Next, we use a pre-trained feature extractor to obtain 1024-dimensional features from each patch and apply a linear projection to compress these features to a more compact 512-dimensional feature vector. These features pass sequentially through four paired Transformer and MCAB modules. Finally, all features are aggregated into a slide-level global representation through gated attention and used for downstream classification tasks. The entire model process can be formulated as:

(3) Z^=MHALayerNormH+H

 

(4) Z=MCABZ^

 

(5) ak=exp⁡wTtanh⁡VzkT⊙sigmUzkT∑i=1Kexp⁡wTtanh⁡VzkT⊙sigmUzkT

 

(6) h=∑k=1Kakzk

The cross-entropy loss function is used to train the model, which can be denoted as:

(7) L=-1N∑i=1NYilog⁡Y^i+1-Yilog⁡1-Y^i

### 3.4 Nystrom attention

Self-attention is the core component of Transformer architectures, designed to address long-range sequence dependencies and has proven to be highly effective. However, the classical self-attention mechanism requires computing pairwise similarities between embeddings, resulting in $O(n^{2})$ time and space complexity, where n represents the number of elements in the input sequence. A typical WSI often contains thousands of patches, making the direct application of self-attention to WSIs computationally prohibitive. To address this computational challenge, we adopt the Nyström attention proposed by (Xiong *et al.* 2021). In this approach, the softmax matrix S in standard self-attention is approximated by the following formula:

(8) S^=softmaxQK∼TdqsoftmaxQ∼K∼Tdq+softmaxQ∼KTdq

Through this method, we reduce the computational complexity of self-attention from $O(n^{2})$ to O(n). This enables us to feed bags composed of thousands of instances into the model.

### 3.5 MCAB

**Figure 3.**
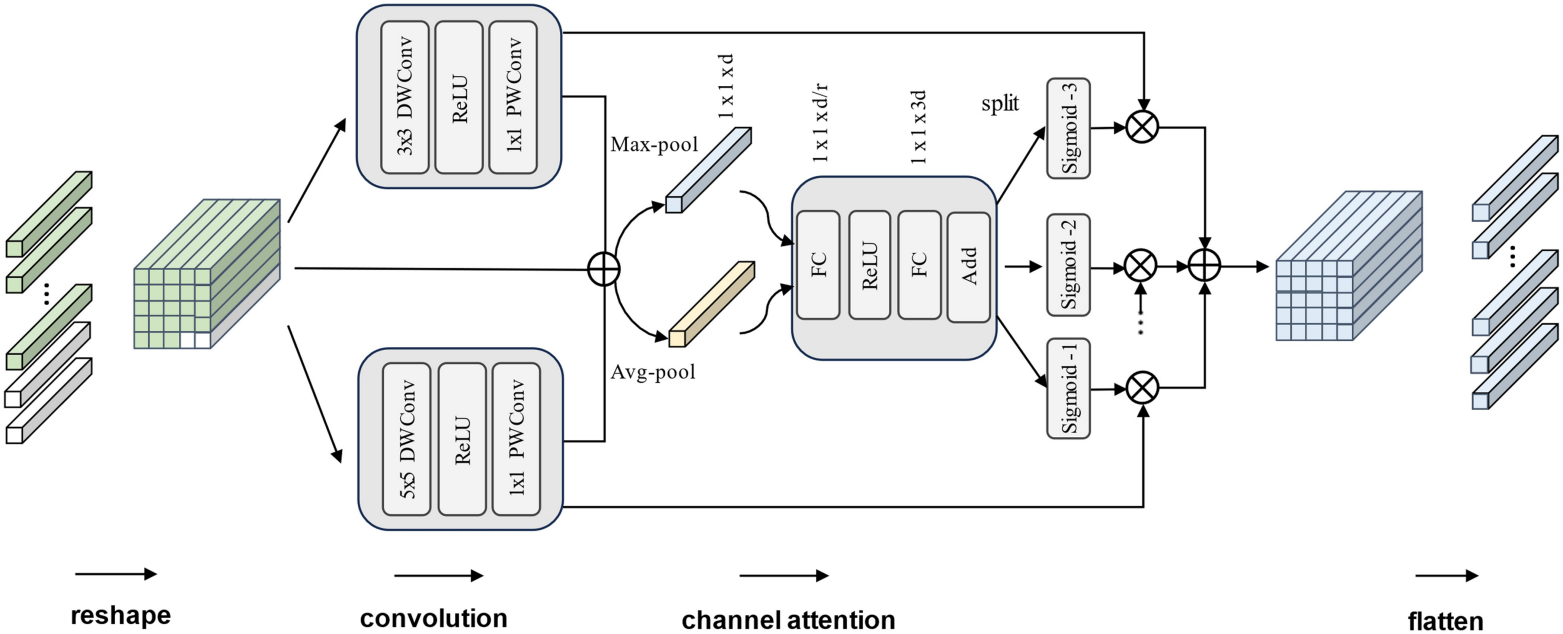
Multi-scale channel attention block. (i) Zero-padding is utilized to reshape 1D sequence to 2D feature map. (ii) Different sized depth-wise separable convolution kernels are used to obtain local texture information and position information. (iii) Channel attention is used to obtain channel-wise weighted feature map. (iv) Feature map is flattened to 1D sequence.

Our primary innovation is the design of a plug-and-play multi-scale channel attention module. This module not only estimates cross-channel relationships at different scales but also provides encoded positional information for the Transformer. Figure 3 shows the internal structure of the MCAB in detail. Specifically, since tokens are ordered in a 1D sequence, traditional 2D convolution operations cannot be applied. Therefore, squaring operation is imperative to reshape the input sequence. However, the number of instances in each bag varies and may not be a perfect square. To facilitate subsequent convolution operations, we first apply a zero-padding operation. In other words, given N instances in a bag, we need to supplement it with $\left(\left\lceil \sqrt{N}\right\rceil \times \left\lceil \sqrt{N}\right\rceil -N\right)$ additional 512-dimensional tokens. Furthermore, these additional tokens only participate in the MCAB computations and not in the Transformer calculations, thereby reducing some computational costs. Squaring operation can be represented as $R^{N\times d}\to R^{M\times M\times d}$, where $M=\left\lceil \sqrt{N}\right\rceil$

Subsequently, we performed the operation of depth-wise separable convolutions (Chollet 2017) to extract local features. We used a pair of depth-wise convolutions of different sizes, each followed by point-wise convolutions. This approach significantly reduces the number of parameters compared to standard convolutions. The local feature extraction process can be denoted as,

(9) Z1=PWConvReLUDWConv1Z^

 

(10) Z2=PWConvReLUDWConv2Z^

After convolution, we sum the original feature map with feature maps obtained from convolutions at different scales. Then, following the approach of CBAM (Woo *et al.* 2018), we utilize max-pooling and average-pooling operations to aggregate the spatial information of the feature maps and obtain two spatial context descriptors. Different from CBAM, our MCAB uses different fine-grained feature maps to capture richer semantic information. Specifically, we first use a shared MLP layer to map above two descriptors from $R^{d}$ to $R^{d/r}$, where r is the scaling ratio for the hidden layer, and then from $R^{d/r}$ to $R^{3d}$. This output is evenly divided into three vectors, each processed with the activation function σ(·) to generate three channel feature maps. We use a sigmoid function with a temperature parameter τ as the activation function, which enhances the sparsity of channel-wise feature selection. Finally, we multiply each of the three channel feature maps with the corresponding feature maps along the channel dimension to enhance important channel-wise features while suppressing information irrelevant to downstream tasks. The whole channel attention process can be depicted in:

(11) L=MLPMaxPoolZ1+Z2+Z^+AvgPoolZ1+Z2+Z^

 

(12) Z=σ1L1⊗Z1+σ2L2⊗Z^+σ3L3⊗Z2

 

(13) σx=11+e-τ·x

The design of CAMIL is inspired by both SE-Net and CBAM. However, SE-Net models only the channel dimension and does not consider the interdependence of the spatial dimension. In addition, although CBAM models both spatial and channel dimensions, it relies on CNNs for spatial dependency modeling, which lack the robust global information capture capabilities of Transformers. To solve above issue, CAMIL uses a Transformer in the spatial modeling stage and uses depth-wise separable convolutions for channel dimension modeling, which significantly reduces the parameter count compared to traditional convolution operations. In addition, SE-Net and CBAM operate on features from a single source, which is insufficient for pathology images rich in texture information. In contrast, MCAB can model channel attention across different fine-grained contextual features simultaneously, enabling the model to capture semantic information more comprehensively.

### 3.6 Evaluation metrics

In this study, accuracy, AUC and F1-score were used as indicators to evaluate model performance. Accuracy represents the ratio of the number of samples correctly predicted by the model to the total number of samples and can be calculated as follows:

(14) Accuracy=TP+TNTP+TN+FP+FN

where TP, TN, FP, and FN represent true positive, true negative, false positive, and false negative, respectively.

AUC represents the area under the ROC curve, which can be used to evaluate the classification effect of the model at different thresholds. According to the physical meaning of AUC, the following calculation formula can be derived:

(15) AUC=∑Ippos>pnegP×N

where $I\left(\cdot \right)$ denotes an indicator function, $p_{\mathrm{pos}}$ is the predicted probability for positive samples, $p_{\mathrm{neg}}$ is the predicted values for negative samples. P indicates the number of positive samples and N indicates the number of negative samples.

F1-score is also a measure of the performance of a binary classification model. It can be treated as a weighted average of precision and recall. The calculation method of F1-score is as follows:

(16) F1-score=2⋅precision⋅recallprecision+recall=2TP2TP+FP+FN

In addition, both Accuracy and F1-score are calculated with 0.5 as the classification threshold.

## 4 Results

### 4.1 Comparison with baseline model

We compared CAMIL with several representative state-of-the-art MIL models (Ilse *et al.* 2018, Li *et al.* 2021a,b, Shao *et al.* 2021, Zhang *et al.* 2022). As demonstrated in Tables 1 and 2, CAMIL achieved superior results across all three datasets.

**Table 1.** Comparison results of CAMIL and other state-of-the-art MIL model on Camelyon16 dataset.

Model	Camelyon16		
	Accuracy	F1-score	AUC
ABMIL	0.8500 ± 0.018	0.8635 ± 0.021	0.9284 ± 0.016
DSMIL	0.8775 ± 0.055	0.8670 ± 0.063	0.9268 ± 0.029
CLAM-SB	0.8575 ± 0.058	0.8662 ± 0.044	0.9211 ± 0.043
CLAM-MB	0.8675 ± 0.044	0.8583 ± 0.048	0.9176 ± 0.026
TransMIL	0.8750 ± 0.038	0.8658 ± 0.046	0.9258 ± 0.046
DTFD	0.8700 ± 0.014	0.8625 ± 0.015	0.9259 ± 0.025
IBMIL	0.8725 ± 0.037	0.8647 ± 0.042	0.9238 ± 0.030
MHIM	0.8725 ± 0.051	0.8370 ± 0.057	0.9126 ± 0.042
CAMIL^a^	0.8800 ± 0.035	0.8736 ± 0.038	0.9512 ± 0.024

a Experimental result of the model.

**Table 2.** Comparison results of CAMIL and other state-of-the-art MIL model on TCGA-NSCLC and TCGA-RCC dataset.

Model		TCGA-NSCLC			TCGA-RCC		
		Accuracy	F1-score	AUC	Accuracy	F1-score	AUC
ResNet50 ImageNet pretrained	ABMIL	0.8304 ± 0.024	0.8700 ± 0.015	0.9064 ± 0.015	0.8784 ± 0.015	0.8523 ± 0.021	0.9718 ± 0.005
	DSMIL	0.8760 ± 0.022	0.8711 ± 0.013	0.9494 ± 0.033	0.8324 ± 0.019	0.8077 ± 0.026	0.9542 ± 0.010
	CLAM-SB	0.8632 ± 0.022	0.8634 ± 0.015	0.9395 ± 0.016	0.8918 ± 0.018	0.8716 ± 0.026	0.9774 ± 0.007
	CLAM-MB	0.8737 ± 0.021	0.8762 ± 0.016	0.9411 ± 0.013	0.9007 ± 0.024	0.8791 ± 0.035	0.9761 ± 0.011
	TransMIL	0.8608 ± 0.028	0.8605 ± 0.011	0.9414 ± 0.022	0.9085 ± 0.022	0.8839 ± 0.041	0.9830 ± 0.002
	DTFD	0.8596 ± 0.017	0.8758 ± 0.010	0.9457 ± 0.017	0.8912 ± 0.047	0.8741 ± 0.030	0.9553 ± 0.012
	IBMIL	0.8678 ± 0.011	0.8687 ± 0.011	0.9423 ± 0.013	0.8941 ± 0.026	0.8730 ± 0.041	0.9772 ± 0.008
	MHIM	0.8760 ± 0.026	0.8760 ± 0.010	0.9495 ± 0.025	0.9084 ± 0.014	0.8909 ± 0.027	0.9787 ± 0.005
	CAMIL^a^	0.8924 ± 0.025	0.8922 ± 0.025	0.9578 ± 0.013	0.9120 ± 0.027	0.8953 ± 0.032	0.9840 ± 0.004
UNI WSI pretrained	ABMIL	0.9345 ± 0.011	0.9335 ± 0.011	0.9773 ± 0.006	0.9415 ± 0.014	0.9412 ± 0.015	0.9767 ± 0.011
	DSMIL	0.9333 ± 0.009	0.9257 ± 0.010	0.9786 ± 0.008	0.9438 ± 0.017	0.9347 ± 0.015	0.9913 ± 0.002
	CLAM-SB	0.9415 ± 0.014	0.9412 ± 0.015	0.9773 ± 0.012	0.9503 ± 0.006	0.9397 ± 0.007	0.9900 ± 0.005
	CLAM-MB	0.9345 ± 0.023	0.9333 ± 0.023	0.9752 ± 0.009	0.9429 ± 0.022	0.9363 ± 0.020	0.9922 ± 0.003
	TransMIL	0.9216 ± 0.017	0.9216 ± 0.017	0.9702 ± 0.012	0.9526 ± 0.014	0.9461 ± 0.014	0.9898 ± 0.002
	DTFD	0.9228 ± 0.013	0.9228 ± 0.013	0.9644 ± 0.023	0.9315 ± 0.013	0.9393 ± 0.029	0.9723 ± 0.014
	IBMIL	0.9240 ± 0.008	0.9239 ± 0.008	0.9726 ± 0.009	0.9416 ± 0.012	0.9352 ± 0.008	0.9906 ± 0.002
	MHIM	0.9287 ± 0.018	0.9286 ± 0.018	0.9747 ± 0.012	0.9438 ± 0.017	0.9347 ± 0.015	0.9913 ± 0.002
	CAMIL^a^	0.9532 ± 0.008	0.9524 ± 0.009	0.9845 ± 0.008	0.9527 ± 0.016	0.9477 ± 0.022	0.9935 ± 0.002

a Experimental result of the model.

Table 1 shows the performance of WSIs classification on the Camelyon16 dataset using the ImageNet-pretrained ResNet50 encoder. It is evident that CAMIL outperforms other models across all three metrics, achieving 2.46% higher in AUC compared to the second best method.

Table 2 shows the performance on the TCGA-NSCLC and TCGA-RCC datasets using the ResNet50 encoder and the UNI encoder (Chen *et al.* 2024). Consistently, CAMIL outperforms other models across multiple metrics, both in binary and multi-class tasks. It is noteworthy that, thanks to the powerful feature representation capability of the foundation model UNI, the performance of all models significantly improved compared to the ImageNet-pretrained ResNet50 extractor.

### 4.2 Effects of MCAB

We conducted ablation experiments on the MCAB from two perspectives: (i) comparing the impact of different scales of depth-wise convolutions on model performance. (ii) Validating the necessity of incorporating point-wise convolutions.

In the MCAB module, we utilized two different convolution kernel sizes, each implying different receptive fields. To explore the most appropriate combination of convolution kernels for CAMIL, we compared three different combinations: 3 × 3 with 5 × 5, 3 × 3 with 7 × 7, and 5 × 5 with 7 × 7. In addition, we removed the pointwise convolution to investigate its contribution to the model's performance.

**Figure 4.**
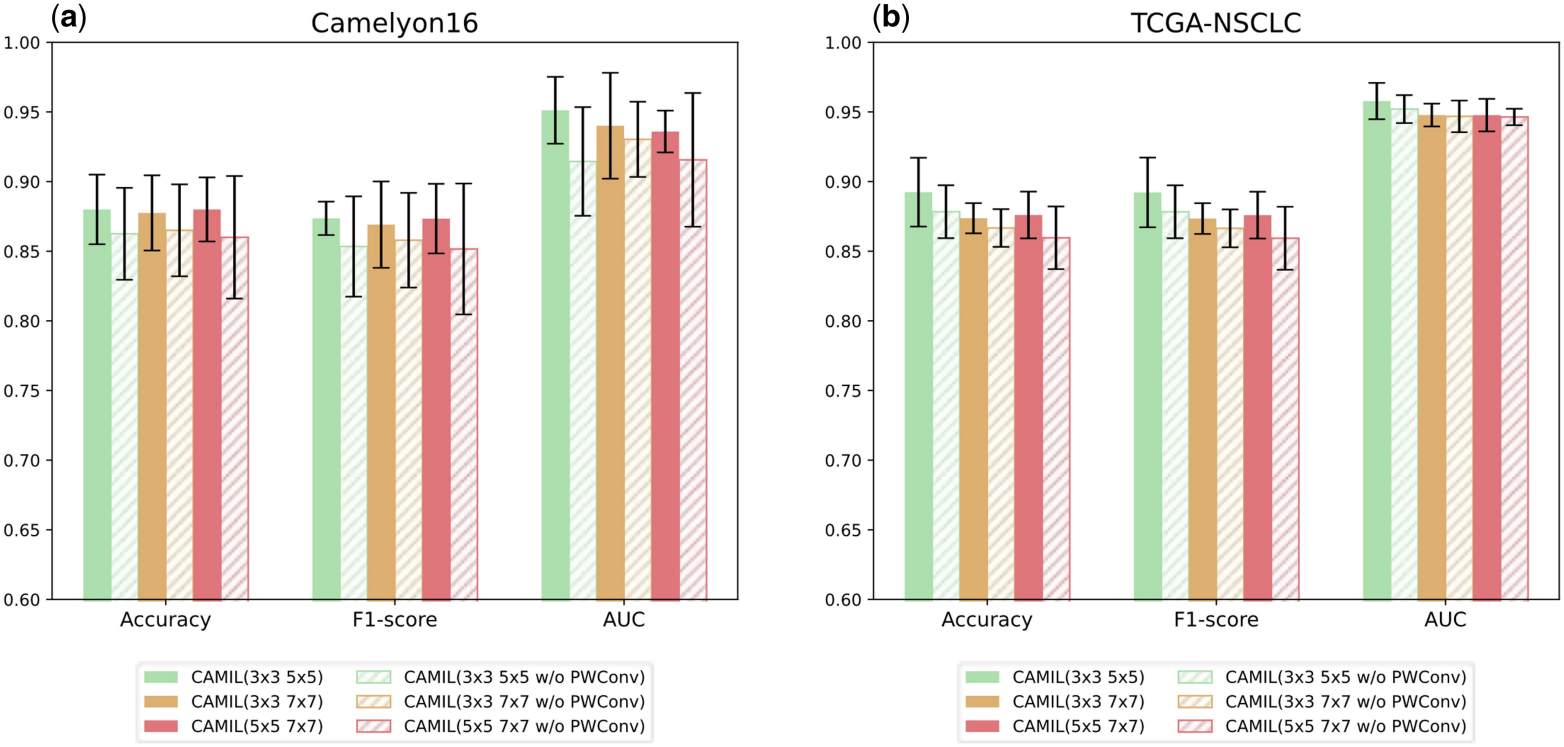
(a) Results of convolution kernel ablation experiments on Camelyon16. (b) Results of convolution kernel ablation experiments on TCGA-NSCLC. **“3 × 3 5 × 5”** indicates sizes of the two depth-wise convolution kernel used by MCAB. **“w/o PWConv”** indicates the removal of the point-wise convolution used in MCAB.

As shown in Fig. 4, introducing point-wise convolution consistently improved model performance across all depth-wise convolution combinations on the Camelyon16 dataset, with an average AUC increase of 0.28%. In contrast, on the TCGA-NSCLC dataset, point-wise convolution provides almost no benefit to the model. Moreover, we observed that the 3 × 3 with 5 × 5 depth-wise convolution kernel combination outperformed other scale combinations. We infer that smaller convolution kernels are more likely to capture the rich texture information contained in the patch.

### 4.3 Effects of input tokens order

**Figure 5.**
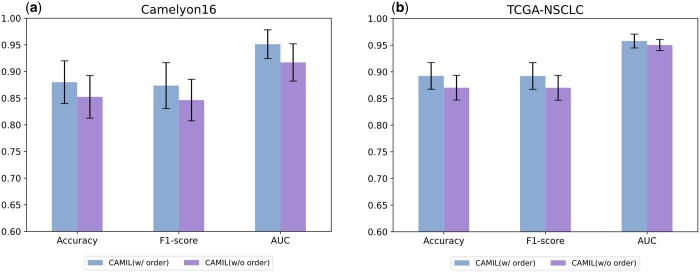
(a) Results of input tokens order ablation experiments on Camelyon16. (b) Results of input tokens order ablation experiments on TCGA-NSCLC. “**w/order”** means tokens are input into the model in order and **“w/o order”** means tokens are input without any specific order.

To validate whether MCAB effectively serves as positional encoding and enhances model performance, we randomized the order of input tokens. As illustrated in Fig. 5a, this disordering of inputs resulted in a significant performance decrease on the Camelyon16 dataset, with all three metrics declining by over 3.4%. Meanwhile, on the TCGA-NSCLC dataset, the three metrics for disordered inputs decreased by an average of 2%. Given that the TCGA-NSCLC training set contains approximately twice the number of samples as Camelyon16, we posit that the rich training data in the TCGA-NSCLC may have mitigated the adverse effects of lacking positional encoding. Moreover, we also evaluated the performance of token sorting in both 1D and 2D arrangements, with the test results shown in Supplementary Fig. S1.

### 4.4 Effects of different pooling operations

In this section, we evaluate the impact of different pooling strategies on model performance through ablation experiments. Pooling layers play a crucial role in deep learning models, particularly when processing sequential data, by reducing feature dimensionality while preserving essential information. We investigated four common pooling methods: class token pooling, max pooling, mean pooling, and attention pooling.

**Figure 6.**
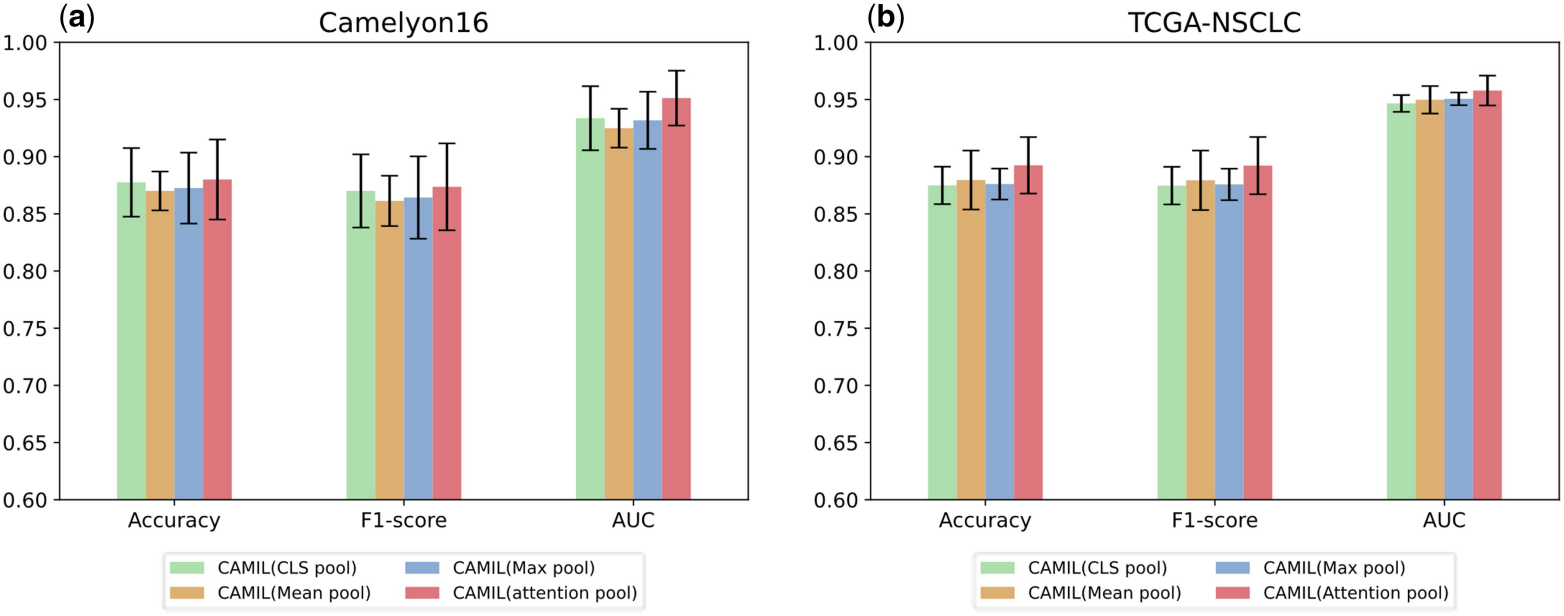
(a) Comparison results of different pooling strategies on the Camelyon16 dataset. (b) Comparison results of different pooling strategies on the TCGA-NSCLC dataset.

To systematically assess the influence of these four pooling methods on model performance, we conducted experiments by replacing the pooling layer preceding the model classifier, maintaining identical model architecture and hyperparameter settings. As illustrated in Fig. 6, on the Camelyon16 dataset, attention pooling performs best, with an average AUC increase of 2.3% over the other three pooling methods. Consistent with previous observations, there is no significant difference among the four pooling methods on the TCGA-NSCLC dataset.

### 4.5 Stress test

**Figure 7.**
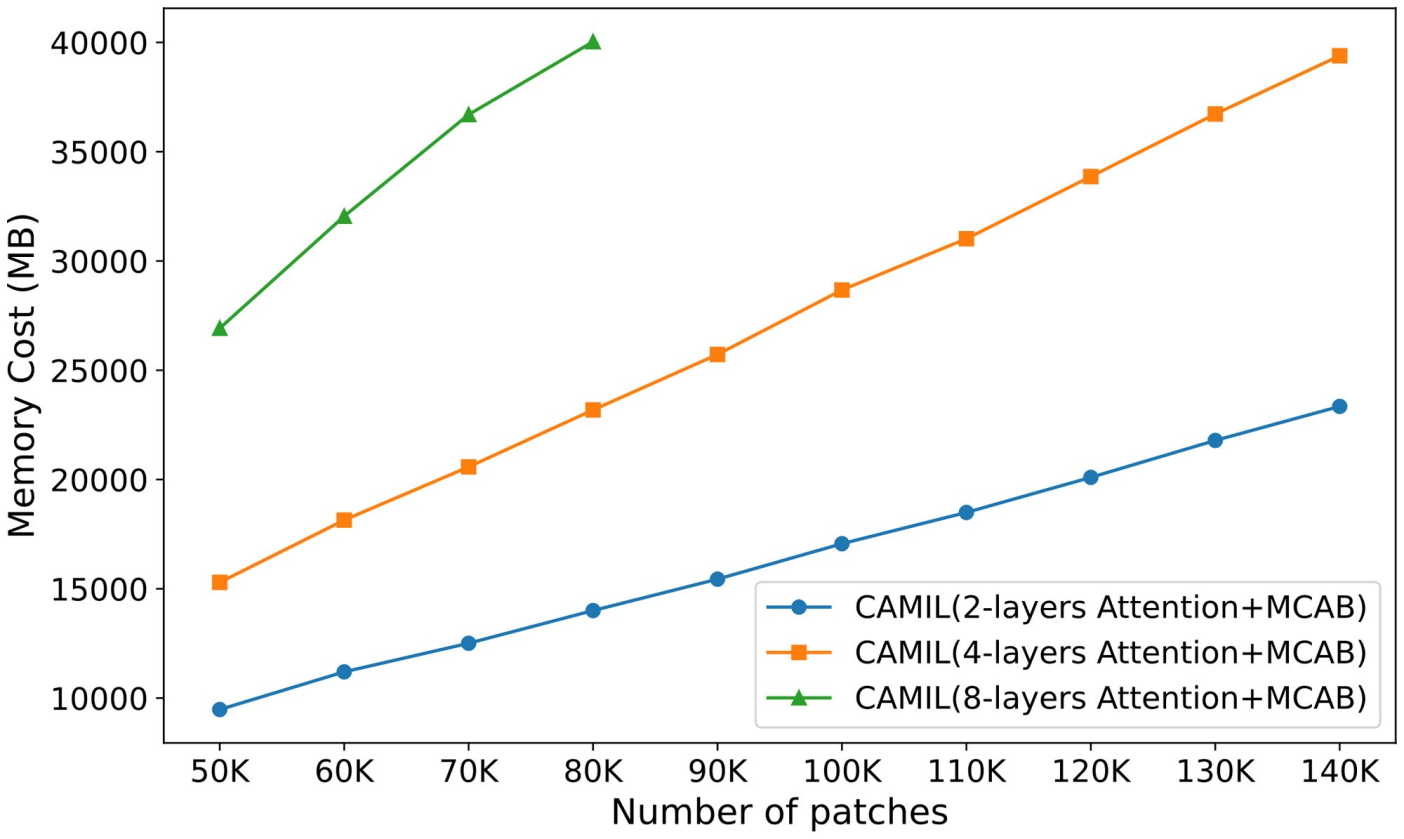
Stress test results of various CAMIL variants on simulation data.

WSIs typically contain a large number of instances, with the number of instances proportional to the area of the tissue region. To assess the computational cost of the model, we generated a series of simulated data for stress testing. Our test environment was an NVIDIA A100 GPU with 40 GB of memory, and all model hyperparameters were kept consistent. Given that the feature aggregator is the primary component of CAMIL, we tested three different variants of the model: 2-layer, 4-layer, and 8-layer Attention-MCAB combination modules. As shown in Fig. 7, it is obvious that the memory usage for all variants is proportional to the number of instances. With the 4-layer configuration, up to 140 000 instances can be processed at once, while the 2-layer and 8-layer configurations can handle up to 80 000 and 240 000 instances, respectively.

### 4.6 Model visualization

**Figure 8.**
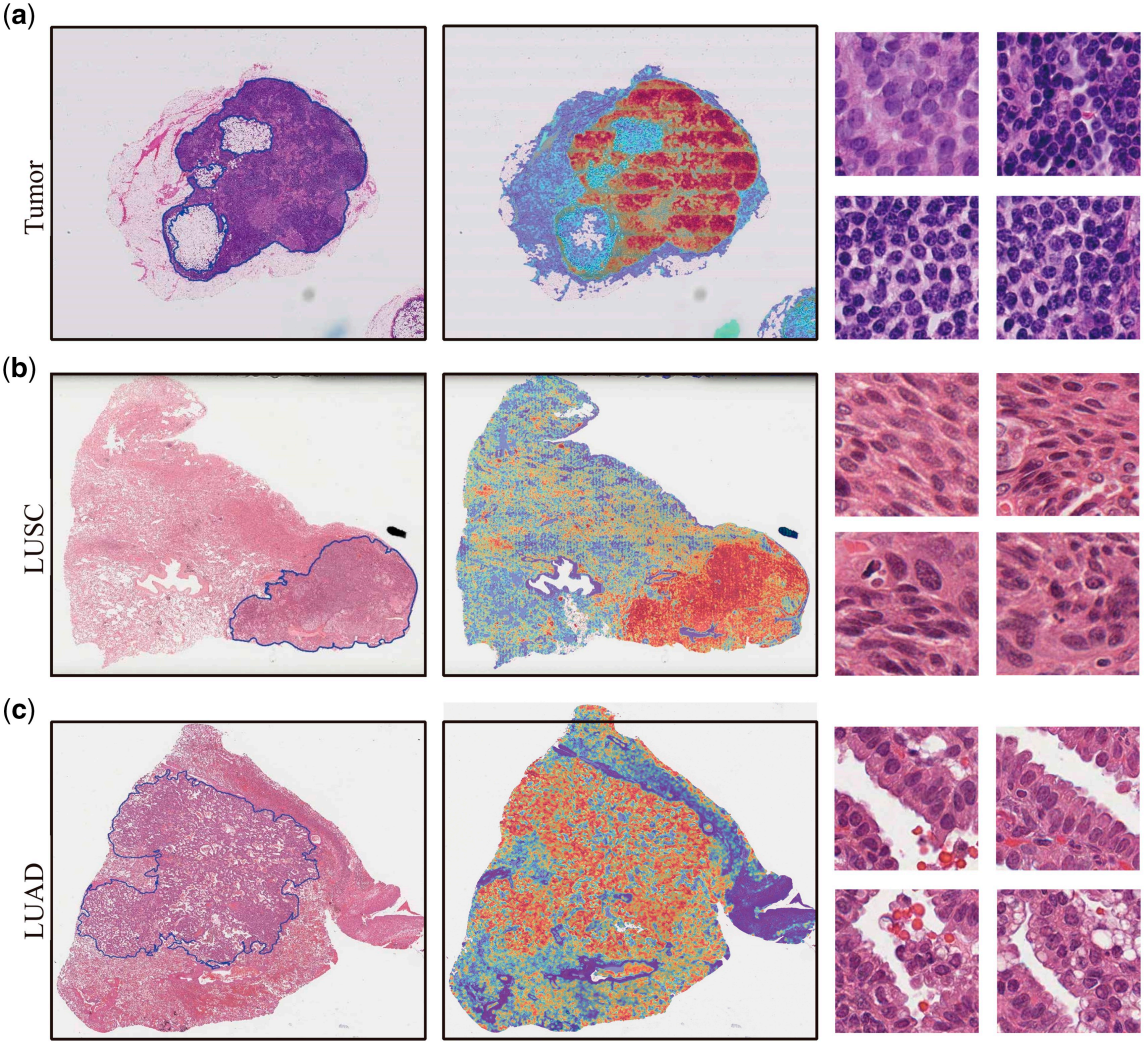
Model visualization and interpretability: on the left is the original slide annotated by a pathologist, in the middle is the heatmap generated by CAMIL’s attention pooler, and on the right is the patch with the highest attention score. (a) is a representative tumor-labeled slide selected from the Camelyon16 dataset, where highlight regions in the heatmap indicate areas associated with lymph node metastasis. (b) and (c) are representative slides of lung squamous cell carcinoma and lung adenocarcinoma, respectively, selected from the TCGA-NSCLC dataset, with highlight regions in the heatmap indicating areas highly relevant to each subtype.

Visualization offers an intuitive approach to presenting complex data and model performance. We selected a slide labeled as tumor from the Camelyon16 dataset and two slides labeled as LUAD and LUSC from the TCGA-NSCLC dataset for visualization. Specifically, the attention scores from CAMIL’s attention pooler were mapped to the corresponding patches in the WSIs. As shown in Fig. 8, regions of interest (high attention scores) are highlighted in red, while areas ignored by the model (low attention scores) are shown in blue. The slides were annotated by an experienced pathologist, and it is easy to observe that the model's focus regions closely align with the annotated regions, demonstrating the model's strong interpretability. To further understand and interpret the key regions the model focuses on, we selected the patches with the highest attention scores for study. On the right side of Fig 8a is a patch highly associated with lymph node metastasis in breast cancer, displaying nuclear pleomorphism, hyperchromasia, a high nuclear-to-cytoplasmic ratio, prominent nucleoli, dense cell arrangement, relatively low stromal components, disorganized arrangement, and blurred cell boundaries. Figure 8b shows a patch highly associated with lung squamous cell carcinoma, where irregular cell arrangement, a high nuclear-to-cytoplasmic ratio, nuclear heterogeneity, unclear cell boundaries, and intercellular bridges can be observed. Figure 8c shows a patch highly associated with lung adenocarcinoma, revealing columnar glandular structures, enlarged nuclei, coarsened chromatin, and nuclear atypia. These patches are highly representative, further indicating the model's ability to classify slides based on identifying key regions.

**Figure 9.**
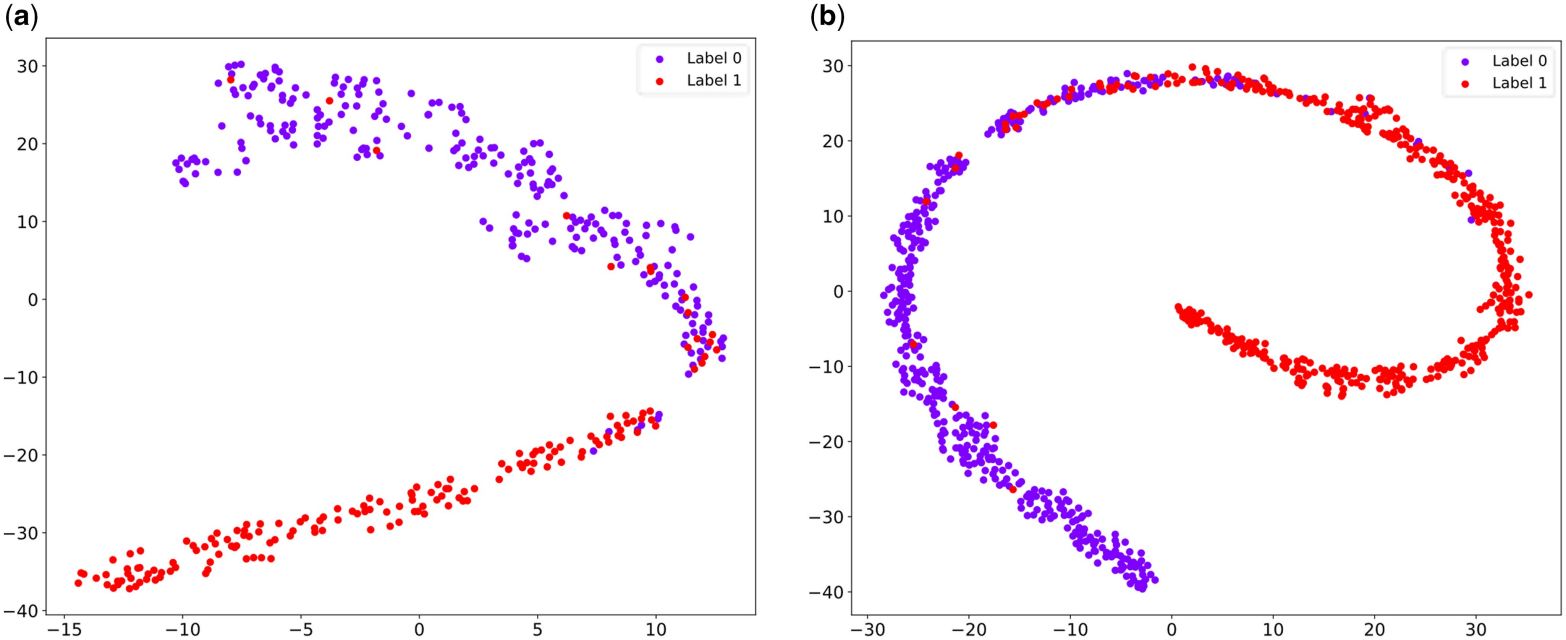
t-SNE visualization of slide-level representation. Dots of different colors represent slides with different labels. (a) is the result of visualizing the slide-level representation of CAMIL on Camelyon16. (b) is the result of visualizing the slide-level representation of CAMIL on TCGA-NSCLC.

We also used t-SNE to visualize the global representations of slides across different categories. On the Camelyon16 dataset (Fig. 9a), the data points display an arc-like distribution with no significant overlap between the two classes, indicating that the data are separable in the feature space. On the TCGA-NSCLC dataset (Fig. 9b), the two categories show clear separation in most regions, with a small overlapping area, suggesting some similarity between these categories in the feature space. This clustering pattern in the visualization demonstrates that the model effectively learns features that distinguish different categories of WSIs.

## 5 Discussion

In this study, we propose a novel method called CAMIL by introducing channel attention into MIL. To better capture the rich texture features in WSIs, we designed a multi-scale channel attention module that generates channel attention maps from feature maps at different granularities. By stacking four Transformer-MCAB modules in sequence, CAMIL is capable of simultaneously model spatial and channel dimensions. Experimental results demonstrate that CAMIL outperforms existing multiple instance methods across various metrics. In addition, we visualized the model using attention maps and t-SNE, further verifying that our model can learn useful information from complex patterns for downstream tasks.

There are still some opportunities for growth in this study. Firstly, due to the limitations of the experimental environment, we conducted experiments using 20× magnification data, where higher magnifications imply significantly more instances. In future work, we plan to further explore the impact of different WSI magnifications on model performance. Secondly, the datasets used in this study have already been well-studied, and there is a lack of validation on real-world data. In future work, we will continue to explore the performance of CAMIL in more clinically meaningful tasks (e.g. predicting immune therapy response). Finally, this study only used a single modality, without integrating other modalities (e.g. cellular or clinical levels), which we plan to study in subsequent research.

## Supplementary Material

btaf024_Supplementary_Data

## Data Availability

The data underlying this article are available in https://portal.gdc.cancer.gov/ and https://camelyon16.grand-challenge.org/Download/.
