# Supplementary material for: CAMIL: channel attention-based multiple instance learning for whole slide image classification
Source: Bioinformatics. 2025 Jan 16;41(2):btaf024. doi: 10.1093/bioinformatics/btaf024 (PMC11802473; doi:10.1093/bioinformatics/btaf024)
Supplement: btaf024_Supplementary_Data [file btaf024_supplementary_data.zip › 6693c_Supplementary.pdf]

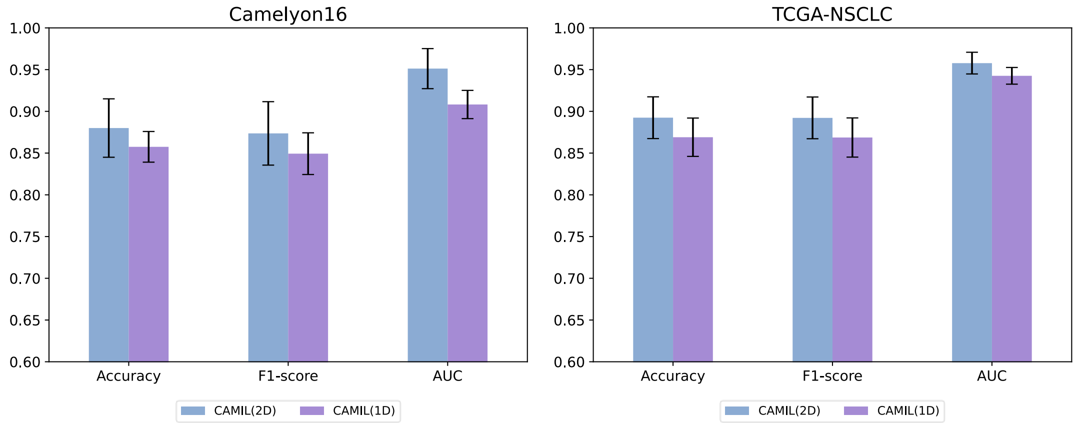

(a) (b)

Fig. S1. Results of input tokens shape ablation experiments on Camelyon16 and TCGA-NSCLC. “**1D”** means tokens are arranged in a 1D manner and **“2D”** means tokens are arranged in a 2D manner.
